# LegitPhish: A large-scale annotated dataset for URL-based phishing detection

**DOI:** 10.1016/j.dib.2025.111972

**Published:** 2025-08-13

**Authors:** Rachana S. Potpelwar, U.V. Kulkarni, J.M. Waghmare

**Affiliations:** Shri Guru Gobind Singh Institute of Engineering and Technology, Vishnupuri, Nanded 431606, Maharashtra, India

**Keywords:** Phishing URLs, Legitimate URLs, Open Phish, Machine learning, Cybersecurity, UCI repository

## Abstract

Phishing attacks are a major cybersecurity threat, requiring timely detection using reliable datasets. We present LegitPhish, a novel and manually verified dataset of phishing and legitimate URLs, designed to facilitate research in machine learning-based phishing detection. LegitPhish, a publicly available dataset of 101,219 labelled URLs, including 63,678 phishing and 37,540 legitimate entries. All URLs are manually verified and annotated with 17 structural and lexical features such as URL length, token count, entropy, subdomain usage, and TLD characteristics. Phishing URLs were collected from threat intelligence feeds (URL Haus, Phish Tank) [1,2] and verified for accuracy, while legitimate URLs were sourced using Google Search API and curated from high-authority domains like Wikipedia. LegitPhish enables reproducible research and benchmarking for phishing detection models and web security applications.

Specifications Table

**Table:** 

Subject	Computer Sciences
Specific subject area	Artificial Intelligence, Cyber Security*.*
Type of data	Raw: csv file*.*
Data collection	Malicious URLs are collected from URL Haus and verified phishing repositories. Legitimate URLs gathered from Stack Overflow and Wikipedia to ensure variety and safety.
Data source location	Worldwide *.*
Data accessibility	Repository name: [Mendeley Repository.][1] Data identification number: *(*10.17632/hx4m73v2sf.2.*)* Direct URL to data: https://data.mendeley.com/datasets/hx4m73v2sf/2
Related research article	None.

## 1 Value of the Data

• This dataset is one of the largest publicly available phishing datasets with over 100,000 URLs and 18 engineered features for machine learning.
• It is valuable for cybersecurity researchers, data scientists, and developers building phishing detection systems.
• Compared to earlier phishing URL datasets, the LegitPhish Dataset offers several advantages:
  – It contains a larger volume of URLs (101,219), providing a more comprehensive dataset for training and evaluation.
  – The dataset includes both phishing and legitimate URLs with a balanced and diverse representation, enabling robust supervised learning.
  – It offers 18 well-engineered features extracted from URL structure, content, and domain-level data, making it suitable for both traditional machine learning and deep learning models.
  – The dataset has been carefully curated from multiple sources including OpenPhish, UCI, and verified legitimate domains, ensuring a high level of data quality and relevance.

This makes it a strong candidate for benchmarking, phishing detection models and developing new cybersecurity techniques.

• Researchers can use this for benchmarking and comparative analysis of phishing detection algorithms.
• Feature-rich design allows for advanced modelling based on URL structure, entropy, tokenization, and domain properties.
• For enhanced clarity, the data specifications are presented as shown in Table 1.

## 2 Background

Phishing is a widespread cyber threat that uses deceptive URLs to impersonate legitimate websites, aiming to steal sensitive user data. As phishing techniques evolve, developing accurate detection systems has become critical. However, the lack of reliable, large-scale, and well-labeled datasets continues to hinder progress in this area. Existing datasets often suffer from issues such as limited diversity, lack of feature richness, or insufficient verification.

The LegitPhish dataset, while designed for building and benchmarking machine learning-based phishing URL detection systems, must also be evaluated in the context of security vulnerabilities. Evasion attacks that exploit model blind spots are a growing threat, especially in real-time detection systems. Works such as ``Artificial Intelligence Security: Threats and Countermeasures'' and ``AI-empowered IoT security for smart cities'' emphasize the necessity of building models resilient to such adversarial manipulations [2,3].

With the increasing integration of phishing detection into secure web platforms and IoT environments, future iterations of LegitPhish may benefit from embedding robust features that defend against model manipulation. Additionally, as the field shifts towards quantum-resistant infrastructures, it is important to consider how LegitPhish-compatible models could incorporate or work alongside post-quantum cryptographic primitives such as Key Encapsulation Mechanisms (KEMs) and signature schemes. NIST's 2023 post-quantum signature competition highlights emerging schemes like Raccoon and NTT that may secure verification or model distribution pipelines [[4], [5]].

The LegitPhish dataset was created to support the development and evaluation of machine learning based phishing detection techniques. It comprises a diverse range of phishing and legitimate URLs, collected from multiple sources to ensure authenticity and balance. Phishing URLs were obtained from verified threat intelligence feeds such as URL Haus and PhishTank, while legitimate URLs were sourced through Google Search API and scraped from reputable websites like Wikipedia and Stack Overflow.

Each URL is accompanied by a set of engineered features that capture lexical, structural, and domain-level characteristics. These include URL length, entropy, token count, subdomain count, presence of IP addresses, and HTTPS usage. The dataset enables reproducible research and facilitates benchmarking for phishing detection models. It supports traditional machine learning as well as deep learning approaches in the field of cybersecurity.

## 3 Data Description

***Data Description*** *the LegitPhish dataset includes 101,219 entries, each labelled as phishing (0) or legitimate (1).*

• Phishing URLs: 63,678
• Legitimate URLs: 37,540

*Each row in the dataset includes the full URL and 17 engineered features. These include lexical, structural, and semantic features that are known to correlate with phishing behaviour.*

***The structure of the standard URL:*** protocol://hostname [: port]/path/ [; parameters] [? query] fragment

## 4 Experimental Design, Materials and Methods

• Phishing Data Collection: Phishing URLs were scraped from URL Haus and other verified phishing repositories using python scripts. Each entry was manually verified or matched against known threat intelligence feeds [6].
• Legitimate URLs: Extracted randomly from popular and reliable websites like Wikipedia and Stack Overflow using custom web scraping tools, ensuring diversity [7].
• Feature extraction: As shown in Table 1, all features were computed using python libraries, including urllib, re, tldextract, and numpy. The entropy was calculated using Shannon’s formula [8].

**Table 1:** Feature description.

Feature Name	Description	Type
URL	Full URL string	Text
url length	Number of characters in the URL	Numeric
has ip address	Presence of IP address (0/1)	Binary
dot count	Number of periods (.) in the URL	Numeric
https flag	HTTPS present (1) or not (0)	Binary
url entropy	Shannon Entropy of the URL string	Numeric
token count	Number of tokens in the URL	Numeric
subdomain count	Number of subdomains	Numeric
query param count	Count of query parameters (e.g.? &)	Numeric
tld length	Length of the top-level domain	Numeric
path length	Length of the URL path section	Numeric
has hyphen in domain	Domain contains a hyphen (0/1)	Binary
number of digits	Total number of digits in the URL	Numeric
tld popularity	Indicator of common TLDs (0/1)	Binary
suspicious file extension	Suspicious file type used (0/1)	Binary
domain name length	Number of characters in the domain	Numeric
percentage numeric chars	% of numeric characters in the URL	Numeric
ClassLabel	Phishing = 0, Legitimate = 1	Binary
• Labelling: Binary labels (0 = phishing, 1 = legitimate) were applied during collection based on source and verification status.

Feature extraction was fully automated via a python script (Algorithm 1), using phishing URLs from the UCI repository, URL haus [9], and PhishTank [10], and legitimate URLs from the Google Search API and Wikipedia. The process was repeated on two URL sets, generating a CSV file of extracted features for easy use with various tools [1].

**Table:** Algorithm 1: Features Extraction Algorithm.

Input: A list of URLs U = {u_1,_ u_2, …….,_ u_n_}
Output: Feature vectors F (u_i_) for each URL u_i_, and their corresponding labels (phishing or legitimate)
1. Steps:
• Initialize on empty list P to store phishing URLs.
• Initialize an empty list L to store legitimate URLs.
2. For each URL u_i_ in U:
• Extract the domain name d_i_ from u_i._
• If uᵢ is found in a verified phishing source (URL Haus, UCI repository, or PhishTank):
• Label uᵢ as phishing.
• Add uᵢ to list P.
• Else if uᵢ is found via Google Search API or from a trusted source (Wikipedia):
• Label uᵢ as legitimate.
• Add uᵢ to list L.
• Otherwise, discard or manually review uᵢ.
3. For each URL uᵢ in the combined list P ∪ L:
• Extract lexical features (URL length, number of digits, token count).
• Extract host-based features (domain name length, subdomain count).
• Extract content-based features (presence of suspicious file extensions, HTTPS usage).
• Calculate entropy of the URL string using Shannon’s formula.
• Combine all extracted values into a feature vector F(uᵢ).
4. Return:
• List of phishing URLs (P)
• List of legitimate URLs (L)
• Feature vectors for all URLs F(uᵢ)

## Limitations

Although this dataset provides a rich and well-balanced resource for phishing URL detection, the dataset primarily contains URLs in English or Latin script domains, which limits its applicability to phishing attacks involving non-English language or region-specific URL patterns future dataset versions could expand linguistic and geographic coverage to reflect global phishing strategies better.

**Fig. 1:**
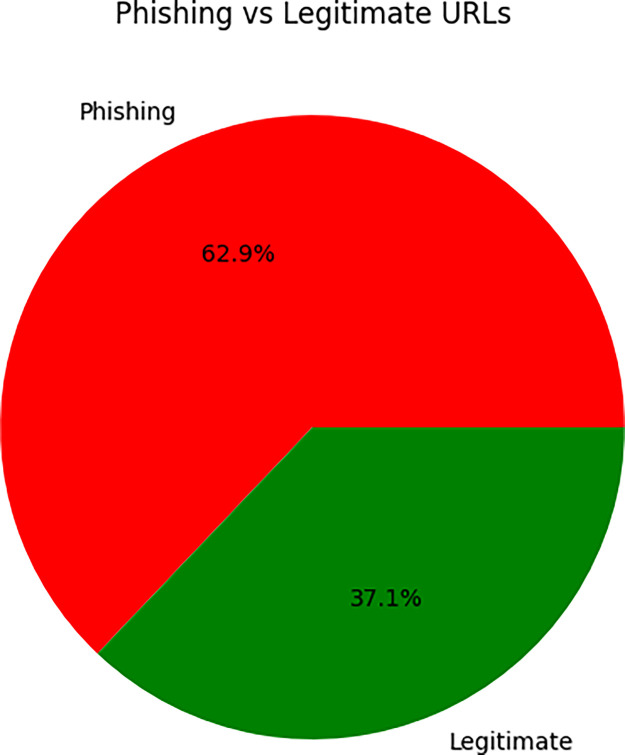
Phishing vs. Legitimate URL Distribution.

The dataset adopts a binary labelling approach classifying each URL strictly as phishing or legitimate (Fig. 1) without accounting for ambiguous or context-dependent cases that may fall into gray areas. These limitations could be overcome by introducing multi-class labels or confidence scores to capture Uncertainty and improve the flexibility of detection models. The dataset currently doesn’t include phishing URLs that have been intentionally modified or disguised to trick detection systems.]

## Future work

• Investigate model robustness against adversarial attacks such as data poisoning and evasion.
• Expand dataset coverage to include multilingual and region-specific phishing attacks.
• Incorporate post-quantum cryptographic primitives for model integrity and secure deployment.

## Ethics statement

The authors declare that they have no known competing financial interests or personal relationships that could have appeared to influence the work reported in this paper.

## Credit Author Statement

**R. S. Potpelwar:** Conceptualization, Methodology, Formal analysis, Data curation, original writing draft**. U. V. Kulkarni:** Investigation, Validation, Visualization, Supervision. **J. M. Waghmare:** Project administration, Supervision, Writing – review, Investigation, Validation.

## Data Availability

Mendeley DataLegitPhish (Original data).
